# Chest pain in a mid-aged woman, not simply myopericarditis: a case report of anti-Ku positive polymyositis

**DOI:** 10.1186/s12872-021-02194-0

**Published:** 2021-08-06

**Authors:** Weiping Tan, Bin Dong, Jincui Gu, Yang Peng, Ruicong Xue

**Affiliations:** 1 Department of Cardiology, the First Affiliated Hospital of Sun Yat-Sen University, 58 Zhongshan Road 2, Guangzhou, 510080 China; 2 NHC Key Laboratory of Assisted Circulation, Sun Yat-Sen University, Guangzhou, China; 3 Department of Respiratory, the First Affiliated Hospital of Sun Yat-Sen University, Guangzhou, China; 4 Department of Radiology, the First Affiliated Hospital of Sun Yat-Sen University, Guangzhou, China

**Keywords:** Myopericarditis, Polymyositis, Anti-Ku, Case report

## Abstract

**Background:**

Anti-Ku is a rare antibody which can be positive in some rheumatic diseases and it might be related to cardiac involvement. Polymyositis is an inflammatory myopathy, and its cardiac involvement seldom presents as myopericarditis and anti-Ku positive.

**Case presentation:**

In this case, we report a mid-aged woman with chest pain, upper limbs weakness and fever unrelated with infection. The diagnosis of this case was unquestionably myopericarditis supported by ECG, cardiac MRI and negative findings in coronary arteries. Diagnosis of polymyositis was further clarified by the evidence of persistently increased CK, degeneration of proximal muscle in MRI, muscular dystrophy with lymphocytes infiltration in muscle biopsy. In the analysis of autoantibodies, we surprisingly discovered positive anti-Ku. Glucocorticoid and mycophenolate mofetil were then prescribed for polymyositis. Patient follow-up indicated remission of both myopericarditis and polymyositis. We finally clarified this rare case as a positive anti-Ku polymyositis with myopericarditis as cardiac involvement.

**Conclusion:**

This report presents a rare case with anti-Ku positive polymyositis and the cardiac involvement of polymyositis was manifested as myopericarditis. Therefore, positive anti-Ku might explain the myopericarditis as cardiac involvement in polymyositis. More cases and longer duration of follow-up is required for the comprehensive understanding of the disease.

## Background

Polymyositis (PM) is one of the subtypes of the inflammatory myopathies, the pathogenesis of which can be attributed to acquired disorder in immune system. Polymyositis is featured as proximal muscle inflammation and extra muscular involvement. Cardiac involvements, mainly manifested as heart failure, myocarditis and arrhythmia, occur in less than 10% of patients with polymyositis [1]. Anti-Ku antibodies are not commonly positive in polymyositis [2]. However, data seems to reveal that prevalence of cardiac involvement in Connective tissue diseases (CTD) might be related to positivity of anti-Ku [3]. However, positive anti-Ku in PM patients with cardiac involvement was rarely studied. Here we report a case of anti-Ku positive polymyositis with cardiac involvement manifested with myopericarditis, which is rarely occurred and reported.

## Case presentation

**Fig. 1:**
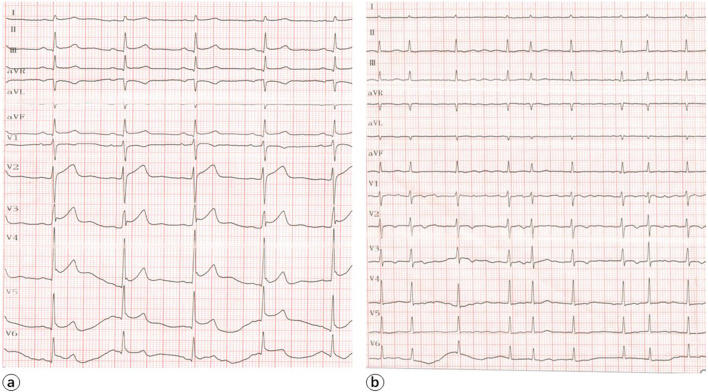
ECG during admission and on the 11th day: **a** ECG at admission showed ST-segment elevation in II/III/AVF and V2-V6 leads; **b** ECG on the 11th day after admission showed recession of ST-segment and new-onset atrial fibrillation

**Fig. 2:**
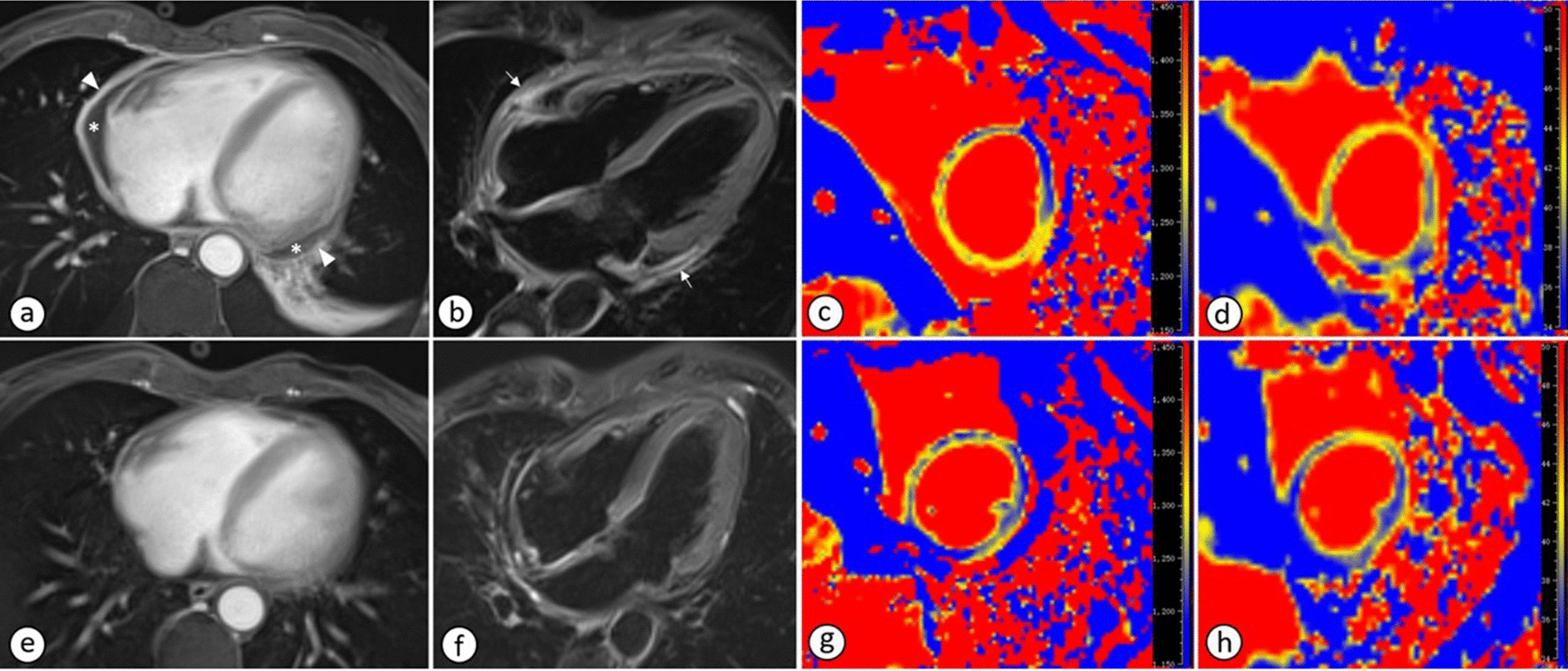
CMR images during episode and follow-up. On axial fat-saturated T1WI with gadolinium enhancement (**a**) and 4-chambered long axis view of fat-saturated T2WI (**b**), diffuse swelling (arrow) and gadolinium enhancement (arrow head) of the pericardium, as well as pericardial effusion (asterisk) were presented. Global native T1- (**c**) and T2-mapping (**d**) were mildly elevated with values of 1300.9 and 41.7, respectively. Follow-up cardiac MR presented diminished pericardial effusion, significantly reduced swelling and enhancement of the pericardium (**e**, **f**). The global native T1- (**g**) and T2-mapping (**h**) value were also decreased with values of 1258.5 and 39.3, respectively

A 56-year-old menopausal female complained with intermittent chest pain for 1 day. The pain was significantly aggravated for 1 h during her shoulder massage. No medication was taken throughout the whole episode. After admission in ER, immediate ECG indicated ST-segment elevation in II/III/AVF and V2-V6 leads and elevated cardiac troponin-I, with sinus rhythm (Fig. 1). 30 min later, emergent coronary angiogram was performed, which showed no obstructive lesions in coronary arteries. Afterwards, she was admitted to cardiac care unit with a primary diagnosis of myopericarditis. Physical examination was remarkable for extremely slender habitus and transient pericardial friction rub, with no obvious crackles. Soon after admission, she presented a new-onset moderate fever (38.6 °C) and atrial fibrillation. Auxiliary examination on the admission day: CRP 55.49 mg/L, ESR 96 mm/h, WBC 18.36 × 109/L, Hb 86 g/L, PLT 192 × 10^9^/L, CK-MB 14.74 ng/mL, MYO 304.80 ng/mL, CK 602U/L, TnT-T 0.215 ng/mL, NT-proBNP 1532.0 pg/mL. Echocardiogram indicated minor pericardial effusion. Up to then, we further confirmed diagnosis of myopericarditis possibly induced by infection. Therefore, the patient was treated with oseltamivir 75 mg twice daily for 6 days, piperacillin tamzotan 4.5 g three times daily for 3 days for anti-infection; intravenous immunoglobulin (IVIg) 20 g once daily for 5 days, coenzyme Q10 and phosphocreatine for myopericarditis; diuretic therapy for heart failure; amiodarone and low molecular heparin for atrial fibrillation. 2 days after admission, ECG showed obvious recession of ST-segment, with alleviated fever and chest pain. Furthermore, the cardiac enzymes including TnT-T, CK, CK-MB gradually declined. However, 10 days after admission, the patient suffered again from a fever with the maximum temperature of 39℃ accompanied with weakness and fatigue. Without positive findings of infection, the patient was empirically treated with meropenem and levofloxacin. However, the fever recurred and the thermal spike remained still. Echocardiogram indicated no neoplasm in valves but enlargement of left ventricle (LVEDD 52 mm) and atria (31 mm * 56 mm * 44 mm) with normal ejection fraction of 54%. Cardiac MR at 3 T (Prisma, Siemens Healthcare, Erlangenm Germany) during this episode indicated diffuse swelling and gadolinium enhancement of the pericardium, as well as pericardial effusion, which was consistent with pericarditis, while elevation of global native T1- and T2-mapping value suggested myocardial edema (Fig. 2). Based on the unusual therapeutic response, we assumed that this patient was not simply myopericarditis due to infection.

**Fig. 3:**
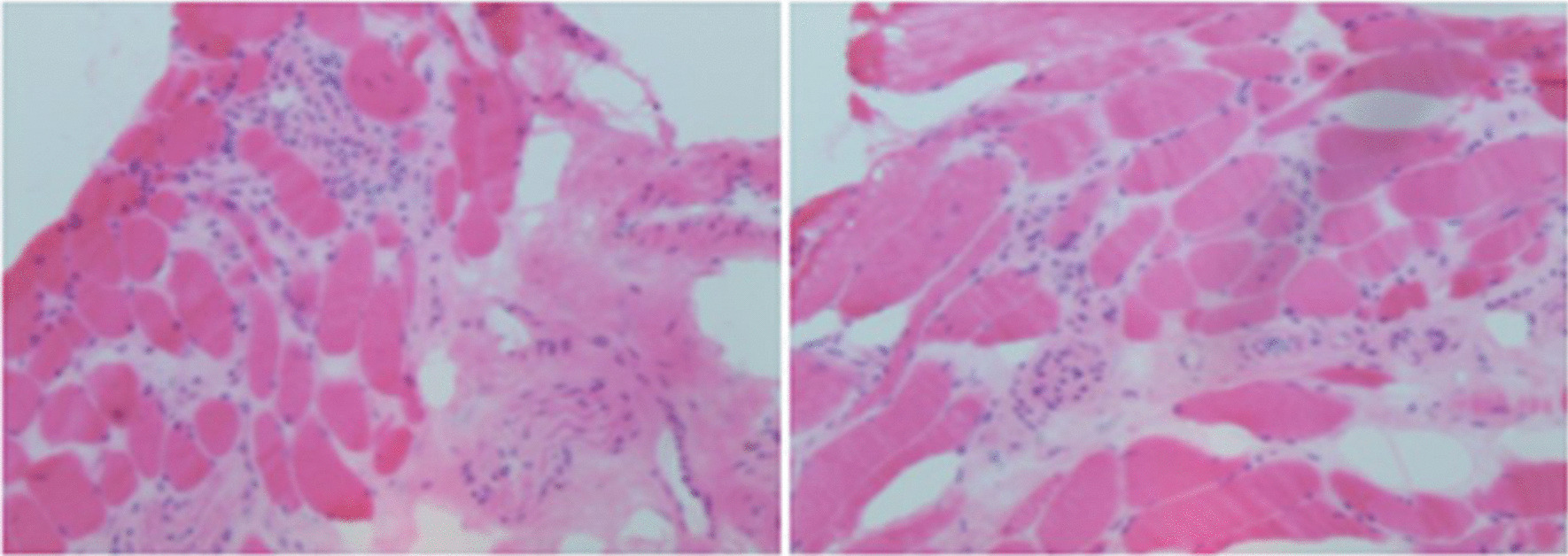
Pathological images of muscle biopsy in the left arm

Importantly, we noticed the mismatched elevation of cardiac enzymes after the episode of fever: CK elevated most (maximum 693U/L, reference range 25–200 U/L), CK-MB (maximum 39 U/L, reference range 0.1–4.94 U/L) and no elevation of TnT-T. Furthermore, we noticed that the ratio of CK/CK-MB in this patient was higher than 10, maximum 67.69. The alteration of CK/CK-MB ratio was not parallel to the alteration of TnT-T. These phenotypes of cardiac enzymes strongly indicated the damage of muscle rather than myocardium. Tracing back, the patient recalled an intermittent pain in her left shoulder-back, accompanied with weakness in upper limbs in the recent 6 months. Myositis was highly suspected based on myodynia and elevated CK. We then explored the evidence of myositis. MRI of bilateral upper arms and shoulder joints indicated degeneration and damage of left supraspinatus, left subscapularis tendon and right supraspinatus tendon, characterized by high signal in T2WI. Abnormal spontaneous activities were discovered in electromyography, indicating myopathic damage. Left arm muscle biopsy showed muscular dystrophy with lymphocytes infiltration (Fig. 3). Based on the above, the patient met the diagnostic criteria of PM according to the Bohan and Peter Criteria [4]. However, the etiology of myositis required further explorations. Negative discovery in PET-CT excluded tumors as the common cause of myositis. Finally, autoantibodies analysis (Table 1) indicated positive anti-Ku IgG (+ + +), anti-Ro-52 IgG (+) and ANA (1:3000). No proteinuria, negative findings in skins and mucosa, negative anti-SS-A, anti-SS-B, anti-Scl-70 etc. excluded myositis secondary to lupus erythematosus, sicca syndrome, systemic sclerosis (SSc), etc. Considering that the myocardium injury in CMR was adjacent to the epicardium, endomyocardial biopsy might not provide positive findings, not to mention its invasiveness. We can conclude the diagnosis of this patient was PM as well as anti-Ku syndrome with myopericarditis as cardiac involvement.

**Table 1:** Autoantibodies in this case

	Results
*Myositis-specific antibodies*	
anti-Jo-1	–
anti-PL-7	–
anti-PL-12	–
anti-EJ	–
anti-OJ	–
anti-Mi-2	–
anti-SRP	–
anti-TIF1-γ	–
anti-NXP-2	–
anti-MDA5	–
anti-SAE	–
anti-HMGCR	–
*Myositis-associated antibodies*	
anti-SSA/Ro	–
anti-Ro52	+
anti-PM-Scl-75	–
anti-PM-Scl 100	–
anti-Ku	+ + +
anti-U1RNP	–
and anti-cN-1A	–

Afterwards, the patient was administrated with methylprednisolone (1 mg/kg/day) 22 days after the initial episode. Fever and fatigue were finally released. 4 days after methylprednisolone administration, CK and CK-MB was reduced to 131U/L and 11.57 U/L, respectively; NT-proBNP returned to normal. The patient was finally discharged. After discharge, the patient was followed up by both cardiologist and rheumatologist. The patient was administrated with continued glucocorticoid and mycophenolate mofetil (MMF) titrated by rheumatologist. The patient complained no upper limb weakness and fever. ESR, CRP, CK, CK-MB levels were gradually decreased and finally normal 4 months after the first glucocorticoid administration. Two months later, a follow-up CMR was performed and demonstrated diminished pericardial effusion, significantly reduced swelling and enhancement of the pericardium. The global native T1- and T2-mapping value were also decreased (global native T1 mapping: from 1300.9 to 1258.5; global native T2 mapping: from 41.7 to 39.3).

## Discussion and conclusions

Our patient was a mid-aged menopausal woman with a chief complaint of acute chest pain, with ST-segment elevation in ECG and elevated TnT-T and CK-MB. Negative discovery in coronary angiogram excluded AMI whereas supported the diagnosis of myopericarditis. Further investigation was not conducted until the patient had an infection-unrelated fever and persisted elevated CK and CK-MB that couldn’t be explained by myopericarditis. Recalling of the pain and weakness of proximal muscle, muscle biopsy changes and muscular damage indications in electromyography met the Bohan and Peter Criteria for PM. Therefore, the patient was diagnosed of PM with myocardium and pericardium involved. Further investigation of the etiology of PM was conducted and PM secondary to tumor and other rheumatic diseases. Strong positive of anti-Ku finally attributed all of the clinical manifestation to anti-Ku syndrome. Anti-rheumatic therapy including glucocorticoid and MMF showed significant therapeutic effect in both PM and PM-related myopericarditis.

### PM with cardiac involvement

PM is one of the types of idiopathic inflammatory myopathies (IIM). It is characterized by gradual progression weakness or pain in proximal muscles, mainly deltoids and hip flexors. PM has been recognized as a systemic disease with multiple organs damaged. Skin, joint, cardiac, gastrointestinal and pulmonary systems manifestations in PM are reported with adverse prognosis, the severity of which might be independent of the progression of PM [5–7]. Cardiac involvement is not rare in PM with the incidence of 9–72% in different studies while is often neglected. Mechanically, cardiac involvement in PM is considered to be sustained inflammation in any structure of the heart, including valves, conduction system, myocardium, pericardium and coronary arteries. Heart failure, myocardial infarction and arrhythmias were the top three cardiac involvements due to PM. The cardiac involvement can be present in any stages of PM, even when PM is in remission [8–10]. The cardiac involvement in our PM patient is both myocardium and pericardium, which is rarely present in PM.

Myositis-specific antibodies (MSA) and myositis-associated antibodies (MAA) detections are necessary in PM. MSAs (such as anti-synthetase antibody, anti-MDA5 and anti-SRP, et al.) are exclusively positive in IIMs and associated with certain clinical features. For example, positive anti-MDA5 indicated intestinal lung disease (ILD) and poor outcome of PM [11]. MAAs (such as anti-Ro52, anti-Ro60, anti-La, anti-U1RNP, PMScl and anti-Ku) are commonly positive in IIM and other rheumatic diseases, especially in overlap syndromes [12]. However, few MSA/MAA are proved to be tightly related with cardiac involvement [13].

### Positive anti-Ku with cardiac involvement

Anti-Ku antibody is a rare MSAs that seldom demonstrated, the clinical meaning of which has not be fully explicated. Unlike other MSAs, anti-Ku cannot be used to stratify the subgroup of diseases due to the lacking evidence of it. A meta-analysis included 9 articles reporting 198 anti-Ku positive patients regardless of their primary diseases, and the study population was involved was any type of CTD, SSc and myositis. The total prevalence of cardiac involvement was 23%. However, the definition of cardiac involvement in the studies above was various and myocarditis was only present in the population of SSc rather than myositis [14]. In a word, there is no anti-Ku positive myositis patient with cardiac involvement presenting with the clinical manifestation of myocarditis in these studies.

Lionel et al. firstly report anti-Ku syndrome defined as positive anti-Ku. They followed up 42 anti-Ku positive patient and analyzed their clinical phenotypes. They demonstrated that two significant phenotypes of anti-Ku positive patients according to their clinical manifestations: Anti-Ku patients with elevated CK, anti-Ku patients with anti-dsDNA. The former phenotype had a higher risk of ILD while the latter one had a higher risk of glomerulonephritis. In his cohort, only 1 out of 42 patient presents with myocarditis, and this patient fell in the cluster of anti-Ku patients with elevated CK [15]. Therefore, we luckily reported this rare case, namely, an anti-Ku PM patient with myopericarditis as cardiac involvement.

Given the rare occurrence of positive anti-Ku in PM with myopericarditis as cardiac involvement, we hope this case report will contribute to the future researches or cohorts in both rheumatic group and cardiovascular group. In addition, longer-duration of follow-up needed to evaluate the effectiveness of anti-rheumatic therapy and the outcome.

## Data Availability

Not applicable.

## Abbreviations

PM: Polymyositis
CTD: Connected tissue diseases
ECG: Electrocardiogram
TnT-T: Cardiac troponin-T
CRP: C-reactive protein
ESR: Erythrocyte sedimentation rate
WBC: White blood cell count
IVIg: Immunoglobulin
CMR: Cardiac magnetic resonance
ANA: Anti-nuclear antibody
SSc: Systemic sclerosis
MMF: Mycophenolate mofetil
AMI: Acute myocardial infarction
IIM: Inflammatory myopathies
MSA: Myositis-specific antibodies
MAA: Myositis-associated antibodies
ILD: Intestinal lung disease
